# Combined effects of targeted blood pressure, oxygenation, and duration of device-based fever prevention after out-of-hospital cardiac arrest on 1-year survival: post hoc analysis of a randomized controlled trial

**DOI:** 10.1186/s13054-023-04794-y

**Published:** 2024-01-12

**Authors:** Martin A. S. Meyer, Christian Hassager, Simon Mølstrøm, Britt Borregaard, Johannes Grand, Benjamin Nyholm, Laust E. R. Obling, Rasmus P. Beske, Anna Sina P. Meyer, Ditte Bekker‑Jensen, Matilde Winther-Jensen, Vibeke L. Jørgensen, Henrik Schmidt, Jacob E. Møller, Jesper Kjaergaard

**Affiliations:** 1 Department of Cardiology, The Heart Center, Rigshospitalet, Copenhagen University Hospital, Blegdamsvej 9, 2100 Copenhagen, Denmark; 2 Department of Clinical Medicine, University of Copenhagen, Copenhagen, Denmark; 3 Department of Anesthesiology and Intensive Care, Odense University Hospital, Odense, Denmark; 4 Department of Cardiology, Odense University Hospital, Odense, Denmark; 5 Department of Clinical Research, University of Southern Denmark, Odense, Denmark; 6 Department of Data, Biostatistics and Pharmacoepidemiology, Center for Clinical Research and Prevention, Bispebjerg and Frederiksberg Hospital, Frederiksberg, Denmark; 7 Department of Cardiothoracic Anesthesiology, Rigshospitalet, Copenhagen University Hospital, Copenhagen, Denmark

**Keywords:** Out-of-hospital cardiac arrest, Post-resuscitation care, Blood pressure, Oxygenation, Temperature control

## Abstract

**Background:**

The “Blood Pressure and Oxygenation Targets in Post Resuscitation Care” (BOX) trial investigated whether a low versus high blood pressure target, a restrictive versus liberal oxygenation target, and a shorter versus longer duration of device-based fever prevention in comatose patients could improve outcomes. No differences in rates of discharge from hospital with severe disability or 90-day mortality were found. However, long-term effects and potential interaction of the interventions are unknown. Accordingly, the objective of this study is to investigate both individual and combined effects of the interventions on 1-year mortality rates.

**Methods:**

The BOX trial was a randomized controlled two-center trial that assigned comatose resuscitated out-of-hospital cardiac arrest patients to the following three interventions at admission: A blood pressure target of either 63 mmHg or 77 mmHg; An arterial oxygenation target of 9–10 kPa or 13–14 kPa; Device-based fever prevention administered as an initial 24 h at 36 °C and then either 12 or 48 h at 37 °C; totaling 36 or 72 h of temperature control. Randomization occurred in parallel and simultaneously to all interventions. Patients were followed for the occurrence of death from all causes for 1 year. Analyzes were performed by Cox proportional models, and assessment of interactions was performed with the interventions stated as an interaction term.

**Results:**

Analysis for all three interventions included 789 patients. For the intervention of low compared to high blood pressure targets, 1-year mortality rates were 35% (138 of 396) and 36% (143 of 393), respectively, hazard ratio (HR) 0.92 (0.73–1.16) *p* = 0.47. For the restrictive compared to liberal oxygenation targets, 1-year mortality rates were 34% (135 of 394) and 37% (146 of 395), respectively, HR 0.92 (0.73–1.16) *p* = 0.46. For device-based fever prevention for a total of 36 compared to 72 h, 1-year mortality rates were 35% (139 of 393) and 36% (142 of 396), respectively, HR 0.98 (0.78–1.24) *p* = 0.89. There was no sign of interaction between the interventions, and accordingly, no combination of randomizations indicated differentiated treatment effects.

**Conclusions:**

There was no difference in 1-year mortality rates for a low compared to high blood pressure target, a liberal compared to restrictive oxygenation target, or a longer compared to shorter duration of device-based fever prevention after cardiac arrest. No combination of the interventions affected these findings.

*Trial registration* ClinicalTrials.gov NCT03141099, Registered 30 April 2017.

**Supplementary Information:**

The online version contains supplementary material available at 10.1186/s13054-023-04794-y.

## Background

Following out-of-hospital cardiac arrest, severe hypoxic-ischemic brain injury account for most fatalities in patients initially resuscitated but still comatose at hospital admission [1]. In the post-resuscitation phase various treatment modalities have been proposed for improving outcomes [1, 2].

The complex pattern of symptoms faced in the care of resuscitated cardiac arrest patients has been termed post-cardiac arrest syndrome (PCAS) [3]. This syndrome consists of four interacting components, namely the pathology that caused the cardiac arrest, the ischemia–reperfusion injury, as well as cardiac dysfunction, and cerebral dysfunction despite return of spontaneous circulation (ROSC) [3].

Current guidelines provide multiple treatment recommendations for the post-resuscitation phase, based mainly on general principles of critical care as well as principles specific for patients with PCAS. These include recommendations for arterial blood pressure and oxygenation targets, and a recommendation for temperature control [2, 4]. However, little evidence exists to inform these guidelines [2, 4].

The “Blood Pressure and Oxygenation Targets in Post Resuscitation Care” (BOX) trial investigated whether a low versus high blood pressure target, a restrictive versus liberal oxygenation target, and a shorter versus longer duration of device-based fever prevention in comatose patients could improve outcomes [5]. No differences in rates of discharge from hospital with severe disability or dying from all causes at 90 days were found for either of the 3 interventions [6–8]. However, even after hospital discharge, cardiac arrest survivors are at increased risk of adverse outcomes, and the long-term or combined effects of these interventions on all-cause mortality are unknown [9–11]. Accordingly, long-term outcomes of cardiac arrest patients could provide a more robust assessment of possible treatment effects, as even subtle short-term effects, not dissectible in rates of mortality or unfavorable neurologic outcome in the early phase, could result in increased risk of long-term morbidity and mortality. Therefore, the objective of this study is to investigate the individual as well as combined effects of the 3 interventions on 1-year mortality rates.

## Methods

**Fig. 1:**
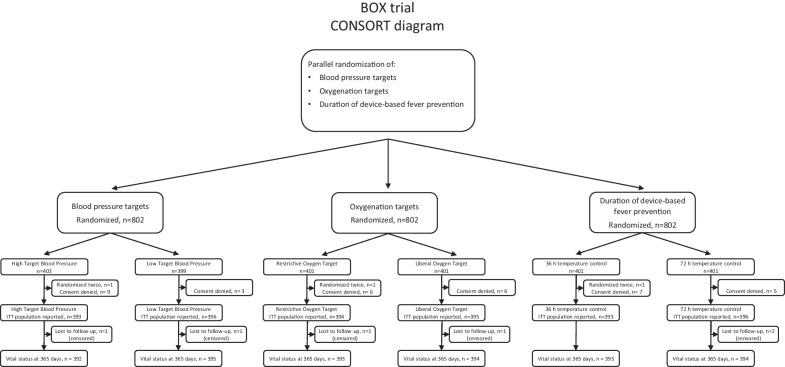
CONSORT diagram for the Blood Pressure and Oxygenation Targets in Post Resuscitation Care (BOX) Trial. Randomization occurred in parallel and simultaneously to the three interventions of blood pressure targets, oxygenation targets, and duration of device-based fever prevention. ITT denotes intention-to-treat population. [6–8] Modified from Kjaergaard et al., N Engl J Med. 2022; Schmidt et al., N Engl J Med. 2022; Hassager et al., N Engl J Med. 2022

In the BOX trial (NCT03141099), comatose resuscitated out-of-hospital cardiac arrest patients were randomly assigned to the following interventions at hospital admission (see Fig. 1): 1. A blinded mean arterial blood pressure target of either 63 mmHg or 77 mmHg; 2. An open-label arterial oxygenation target of 9–10 kPa (68–75 mmHg) or 13–14 kPa (98–105 mmHg); 3. Device-based fever prevention administered as temperature control for the initial 24 h at 36 °C and then either 12 or 48 h at 37 °C, for a total of 36 or 72 h (open-label). The trial was designed as a randomized controlled, two-center trial with a 2 × 2 factorial design for the primary interventions of blood pressure and oxygenation targets, combined with a subordinate randomization for the duration of device-based fever prevention. Approval was obtained prior to trial initiation from the regional ethics committee of The Capital Region of Denmark and handling of patient data had been approved by the Danish Data Protection Agency. The trial is registered at ClinicalTrials.gov NCT03141099; registered with first submission of record 30 April 2017, 11 patients had been included prior to publication of the record. The protocol with trial rationale and statistical plan [5] and the short-term outcomes for the three interventions have previously been published [6–8].

### Patients and randomization

Patients were recruited from 2017 until 2021 at two Danish tertiary hospitals (Copenhagen University Hospital, Rigshospitalet, and Odense University Hospital). The eligibility criteria were patients resuscitated from out-of-hospital cardiac arrest with a presumed cardiac etiology, age ≥ 18, return of spontaneous circulation for at least 20 min, and inability to obey verbal command; assessment of eligibility was assessed as early as possible after hospital admission, and inclusion had to be performed within 240 min from time of ROSC; please see supplement for a full description of in- and exclusion criteria [5]. If all inclusion criteria and no exclusion criteria were met, patients were randomized for all three interventions simultaneously using a secure web-based system. For each intervention, patients were randomized 1:1 to either arms, in random permuted blocks of 2, 4, or 6, and stratified by site.

### Trial interventions

All patients were initially treated in an intensive care unit in accordance with guidelines from the European Resuscitation Council at the time of design and conduct of the trial [12]. As standard of care, all patients were sedated, ventilated, and treated with temperature control at 36 °C for at least 24 h. For the low versus high blood pressure target intervention, a patient specific blood pressure module that had been calibrated to indicate either a 10% higher or lower pressure than actual pressure was used for as long as the patient underwent invasive blood pressure monitoring. By aiming at a mean arterial pressure of 70 mmHg for all patients, this would result in actual targets of either 63 mmHg or 77 mmHg according to whether the patients had been randomized to a low or high blood pressure target, and the intervention was blinded for the clinical staff [13]. For the restrictive versus liberal oxygenation target intervention, patients were assigned to an open-label arterial oxygenation target of either 9–10 kPa (68–75 mmHg) or 13–14 kPa (98–105 mmHg), and the fraction of inspired oxygen (FiO2) was adjusted to reach the assigned targets as soon as possible after randomization and maintained until extubation. For the duration of device-based fever prevention intervention, patients were assigned to an open-label duration target for device-based fever prevention of either 12 or 48 h following the mandatory period of 24 h at 36 °C for all patients. Thus, patients would have a total of either 36 or 72 h of temperature control, dependent on group assignment, and the set temperatures were for all patients initially 36 °C for 24 h, and then 37 °C during the period beyond 24 h. Device-based fever prevention past the initial 24 h was terminated if patients regained consciousness within the intervention period, regardless of group assignment.

### Outcomes

Enrolled patients were followed for 1 year (365 days) for the occurrence of death from all causes. Rates of mortality are reported separately for each of the 3 interventions. This outcome was assessed by means of the electronic medical record which is linked to the national person data registry in Denmark. Assessment of neurologic function at 1 year after randomization, was performed by use of cerebral performance category (CPC) and based on review of medical records [14, 15]. The CPC score ranges from 1 to 5 with lesser scores indicating better performance, and a score of 1 and 2 signifies a favorable neurologic outcome, while scores of 3 to 5 represent a poor neurologic outcome [14, 15].

### Statistics

Data for this combined follow-up study on 1-year mortality according to all three interventions of the BOX trial are based on the intention-to-treat population as were the previously published manuscripts on the primary findings at 3 months [6–8], and outcomes for all patients of this population are reported for all interventions. The outcome of 1-year mortality was analyzed by a Cox proportional model adjusted for site for all three interventions and reported as hazard ratio (HR) with 95% confidence interval and p value for the model. The possible interactions of the interventions were also tested in a cox proportional model adjusted for site. Further, to investigate the combined effects, cox models for each of the three interventions adjusted for site and stratified by co-randomization of the other 2 interventions were performed. Possible interactions of predefined subgroups on 1-year mortality were analyzed by means of a cox proportional models stratified by each of the following separately: sex; age at or above the median or below the median; site; whether the patient had chronic obstructive pulmonary disease; known renal impairment; known hypertension at the time of cardiac arrest; whether primary rhythm was shockable; and whether the ECG after resuscitation from cardiac arrest indicated ST-segment elevation myocardial infarction or not. For assessment of the proportion of patients with a poor compared to a favorable outcome for each of the interventions, we report risk ratios (RR) and 95% confidence intervals. The significance level was set at *p* < 0.05. Statistical analyses were performed in SAS Enterprise Guide version 7.15 (SAS Institute, Cary, NC).

## Results

In the BOX trial, all patients were randomized to all three interventions (Fig. 1), and 789 patients were included in the primary analyses; see Table 1 for patient characteristics. For the blood pressure intervention, 396 patients were randomized to a low blood pressure target and 393 to a high target. For the oxygenation intervention, 394 patients were randomized to a restrictive oxygenation target, and 395 to a liberal oxygenation target. For the device-based fever prevention intervention, 393 patients were randomized to a total duration of temperature control for 36 h and 396 to a total duration of 72 h. For the assessment of 1-year mortality, two foreign participants could not be followed for this endpoint and were censored at the date of last contact; thus, data at 1 year were available for 787 of 789 patients (99.7%). For the assessment of neurologic outcome at 1 year, 18 patients could not be evaluated, and therefore, for this endpoint, data were available for 771 of 789 patients (97.7%).

**Table 1:** Patient characteristics

	Low blood pressure target	High blood pressure target	Restrictive oxygen target	Liberal oxygen target	Temperature control for 36 h	Temperature control for 72 h
n	396	393	394	395	393	396
Age	65 (53–72)	64 (55–73)	63 (53–72)	65 (55–73)	64 (54–72)	64 (54–73)
Female sex	76 (19%)	76 (19%)	70 (18%)	82 (21%)	73 (19%)	79 (20%)
*Previous medical history*						
Ischemic heart disease	78 (20%)	94 (24%)	89 (23%)	83 (21%)	93 (24%)	79 (20%)
Heart failure	72 (18%)	65 (17%)	58 (15%)	79 (20%)	71 (18%)	66 (17%)
Hypertension	186 (47%)	176 (45%)	215 (51%)	210 (49%)	186 (47%)	176 (45%)
Renal impairment	17 (4%)	22 (6%)	19 (5%)	20 (5%)	20 (5%)	19 (5%)
COPD	33 (8%)	30 (8%)	29 (7%)	34 (9%)	26 (7%)	37 (9%)
*Characteristics of the cardiac arrest*						
Bystander CPR	339 (87%)	340 (88%)	346 (89%)	333 (86%)	340 (88%)	339 (87%)
*First monitored rhythm*						
Shockable	350 (88%)	356 (91%)	351 (89%)	355 (90%)	352 (90%)	354 (90%)
Nonshockable	46 (12%)	35 (9%)	42 (11%)	39 (10%)	40 (10%)	41 (10%)
Time to ROSC	18 (12–25)	19 (12–27)	19 (12–25)	18 (12–26)	19 (12–25)	18 (12–26)
*Admission status*						
pH	7.24 (7.16–7.30)	7.23 (7.15–7.29)	7.23 (7.15–7.29)	7.24 (7.16–7.30)	7.23 (7.15–7.29)	7.24 (7.17–7.30)
Lactate, mM	4.9 (2.9–7.4)	5.1 (2.9–8.3)	4.9 (3.0–7.8)	5.0 (2.8–8.0)	5.1 (3.1–8.0)	4.8 (2.7–7.8)
PaO2, kPa	11.3 (7–19.5)	9.8 (6.6–19.5)	10.0 (6.5–19.0)	11 (7.2–19.7)	10.8 (7.2–20.1)	10.3 (6.3–18.8)
Temperature, °C	35.5 (34.8–36.0)	35.5 (34.8–36.1)	35.4 (34.7–36.0)	35.6 (34.9–36.1)	35.6 (34.9–36.1)	35.4 (34.6–36.0)
ROSC at admission	374 (97%)	369 (96%)	371 (96%)	372 (97%)	374 (97%)	369 (95%)
STEMI	178 (47%)	172 (44%)	179 (47%)	171 (44%)	172 (44%)	178 (46%)
Acute CAG	358 (90%)	364 (93%)	359 (91%)	363 (92%)	359 (91%)	363 (92%)
Whereof PCI	165 (46%)	171 (47%)	177 (49%)	159 (44%)	161 (45%)	175 (48%)

Data shown as n (%) or median (interquartile range). COPD denotes chronic obstructive pulmonary disease; CPR, cardiopulmonary resuscitation; ROSC, return of spontaneous circulation; PaO2, arterial partial pressure of oxygen; STEMI, ST-segment elevation acute myocardial infarction; CAG, coronary angiography; PCI, percutaneous intervention. Patient characteristics including additional variables have previously been published [6–8]

**Fig. 2:**
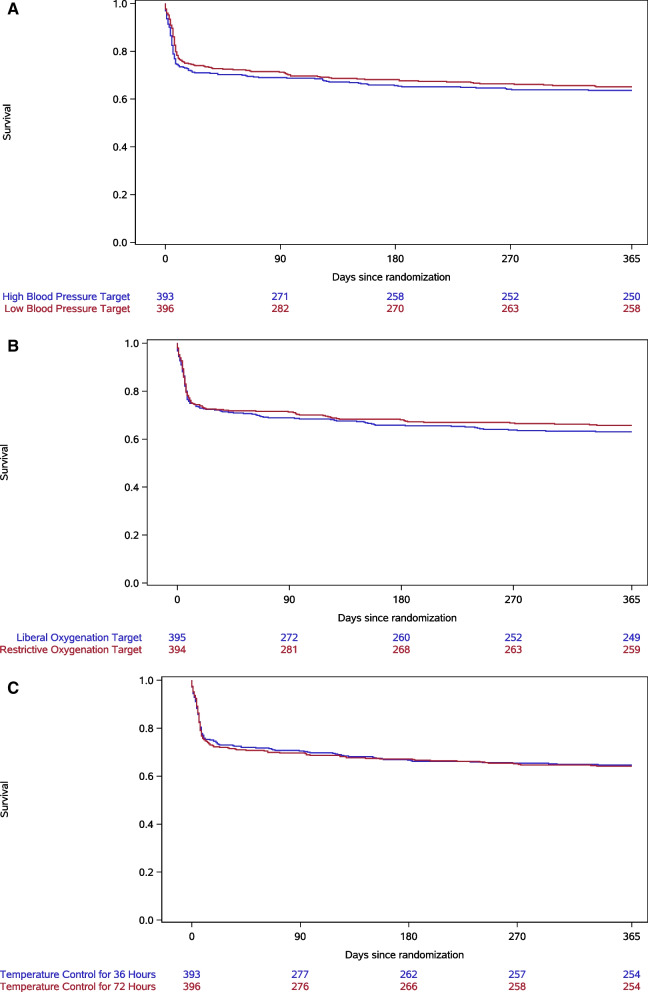
Kaplan–Meier plot of survival for 365 days after out-of-hospital cardiac arrest shown for **A** Low compared to high blood pressure targets, **B** Restrictive compared to liberal oxygenation targets, and **C** Device-based fever prevention for 36 compared to 72 h

The 1-year mortality rates for low compared to high blood pressure targets were 35% (138 of 396) and 36% (143 of 393), respectively (Fig. 2A), adjusted hazard ratio (HR) 0.92 (95% CI 0.73–1.16) *p* = 0.47. For the interventions of restrictive compared to liberal oxygenation targets, the 1-year mortality rates were 34% (135 of 394) and 37% (146 of 395), respectively, adjusted HR 0.92 (0.73–1.16) *p* = 0.46 (Fig. 2B). For the interventions of device-based fever prevention for a total of 36 h compared to 72 h the 1-year mortality rates were 35% (139 of 393) and 36% (142 of 396), respectively, adjusted HR 0.98 (0.78–1.24) *p* = 0.89 (Fig. 2C).

Analyzes of effects of the interventions according to the predefined subgroups did not show evidence of differentiated treatment effects of a low compared to high blood pressure targets, a restrictive compared to liberal oxygenation target, nor a total duration of device-based fever prevention for 36 h compared to 72 h, for neither of the subgroups (see Additional file 1: Figs. S1–3).

**Fig. 3:**
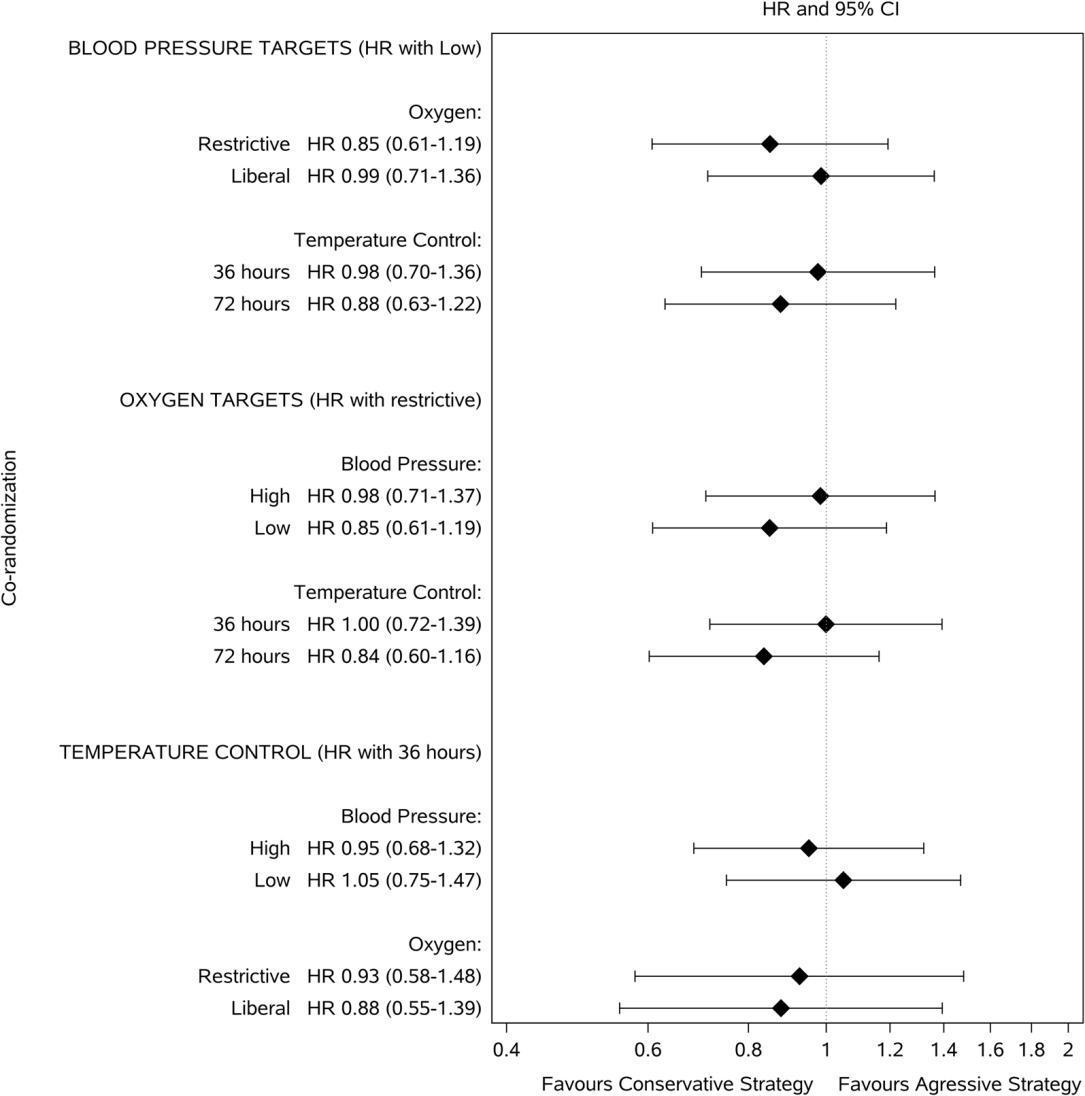
Forest plot showing the three randomizations of low versus high blood pressure target, a restrictive versus a liberal oxygenation target, and a shorter versus longer duration of device-based fever prevention, each stratified by the co-randomizations. An aggressive strategy includes a main randomization to a high blood pressure target, a liberal oxygen target, and temperature control for 72 h. A Conservative strategy includes a main randomization to a low blood pressure target, a restrictive oxygen target, and temperature control for 36 h

There was no interaction between the two primary interventions of blood pressure and oxygenation targets (*p* = 0.55), nor was there an indication of interaction when considering all three interventions combined (*p* = 0.69), and no combination of the interventions indicated differentiated effects of each of the three interventions when stratified according to the other two co-randomizations (Fig. 3).

The proportions of patients with a poor neurologic outcome at 1 year for a low compared to high blood pressure targets were 37% (143 of 385) and 39% (149 of 386), respectively (Additional file 1: Fig. S4), RR 0.96 (0.80–1.15). For restrictive compared to liberal oxygenation targets the proportions of patients with a poor neurologic outcome at 1 year were 36% (139 of 385) and 40% (153 of 386), respectively (Additional file 1: Fig. S5), RR 0.91 (0.76–1.09). For device-based fever prevention for a total of 36 h compared to 72 h the proportions of patients with a poor neurologic outcome at 1 year were 38% (144 of 384) and 38% (148 of 387), respectively (Additional file 1: Fig. S6), RR 0.98 (0.82–1.17).

## Discussion

The present 1-year results of the BOX trial did not indicate differences in mortality rates for neither a low compared to high targeted blood pressure, a liberal compared to a restrictive oxygenation target, or a shorter compared to longer duration of device-based fever prevention. There were no signs of interaction between the three interventions. Analyses of possible differentiated effects based on subgroups did not show evidence of differentiated treatment response according to patient characteristics or circumstances of the cardiac arrest for either of the interventions. Further, there were no differences in proportions of patients with a poor neurologic outcome at 1 year for low compared to high targeted blood pressure, a liberal compared to a restrictive oxygenation target, or a shorter compared to longer duration of device-based fever prevention.

The findings of no observed benefit on rates of 1-year mortality or proportions of patients with a poor neurologic outcome in the present investigation, from a low compared to high blood pressure target during the post-resuscitation phase, are in line with of our previous findings at 3 months [6–8]. The BOX trial is the only larger trial to randomize patients to a low or high blood pressure target, and a recent meta-analysis based on four randomized studies, including the BOX trial, found no benefit of targeting a higher compared to lower blood pressure after cardiac arrest with respect to mortality at 180 days [16]. The analysis of possible differentiated effects in subgroups with respect to the primary endpoint of death at 90 days or discharge from hospital with severe disability, indicated a possible benefit of a high compared to low blood pressure in patients with COPD [6]. There was no indication of such possible differentiated effect at 1-year for any of the subgroups in the present investigation, suggesting that this could have been a chance finding at 3 months. In the present trial, the high mean arterial blood pressure target was 77 mmHg, and the low 63 mmHg [5, 6]. Current guidelines recommend a mean arterial pressure of > 65 mmHg similar to the low blood pressure target in the present trial [2]. While the mean difference of 10.5 mmHg in mean arterial pressure observed in the trial was considered clinically relevant and accompanied by greater vasoactive-inotropy score in the high blood pressure group [6], we cannot rule out that even higher blood pressure targets may be beneficial. This will be addressed by the recently launched STEPCARE randomized trial (NCT05564754) that investigates if an open-label MAP target of > 85 mmHg confers a benefit over a target of > 65 mmHg after out-of-hospital cardiac arrest. While the BOX trial investigated if a higher than guideline recommended blood pressure was beneficial, we cannot rule out that targeting a lower pressure than > 65 mmHg could be beneficial.

Finding the optimum oxygenation target for critical care patients, including patients resuscitated from cardiac arrest, has been the subject of multiple recent trials and meta-analyses [7, 17–19]. The ICU-ROX trial investigated in a broad critical care patient population if a conservative-oxygen therapy, to maintain a saturation above 90% and below 97% using the lowest possible fraction of inspired oxygen conferred a benefit, compared to usual care with no restriction on upper saturation limits or fraction of inspired oxygen [17]. The trial found no overall effect on the primary outcome of ventilator-free-days, and secondarily, there was no overall benefit on mortality at 180 days [17]. A post hoc subgroup analysis for patients with hypoxic-ischemic brain injury due to cardiac arrest did however indicate a lower rate of mortality at 180 days for the conservative compared to the usual oxygen therapy group [17]. This finding was not statistically significant when adjusting for patient characteristics and conditions of the cardiac arrest, when examined in a post hoc substudy with additional available information [20]. In the EXACT-trial published in 2022, it was investigated if patients resuscitated after out-of-hospital cardiac arrest would benefit from lower compared to higher degrees of oxygen supplementation in the immediate aftermath of resuscitation as this was hypothesized to limit brain reperfusion injury [18]. The investigators randomized patients in the field to either a peripheral saturation of 90–94% or 98–100%, and while the trial was stopped early due to the COVID-19 pandemic, it indicated possible harm from the oxygenation target of 90–94%, as fewer patients survived to discharge compared to the target of 98–100% [18]. In the BOX trial, we found no benefit of a restrictive compared to liberal oxygenation strategy at 3 months in patients resuscitated after out-of-hospital cardiac arrest on the primary endpoint of discharge from hospital in a poor neurologic state or death from all causes [7]. A meta-analyses of higher or lower oxygenation targets after cardiac arrest, which included the BOX trial as the largest contributor of patients, also found no difference in survival at 90 days from a higher versus lower oxygenation target [19]. The present investigation extends that finding as there was no indication of a survival benefit at 1 year after cardiac arrest. This could indicate that the PaO_2_ ranges across the restricted and liberal oxygenation targets as used in the BOX trial while patients were in the intensive care unit, are safe. Based on the BOX trial we cannot rule out that more extreme ranges of lower or higher arterial oxygen levels could yield different results [21].

Temperature control after out-of-hospital has been part of contemporary guidelines since 2003 [22]. Initially, temperature control at 32–34 °C was applied for 12–24 h, with no active prevention of fever after this period, and it was restricted to patients resuscitated after out-of-hospital cardiac arrest caused by ventricular fibrillation [22]. In 2005, temperature control was expanded to include fever prevention for a total duration of 72 h after cardiac arrest [23]. This recommendation was not informed by randomized data. Instead, it was founded on observational data indicating an association between poor outcomes and observed fever in the post-resuscitation period [24], and the randomized trials published in 2002 indicating a possible benefit from hypothermia after resuscitation from cardiac arrest [25, 26]. However, since the initial trials on hypothermia after cardiac arrest [25, 26], which both were of limited size and with possible risk for confounding [27], two major trials—the TTM and TTM 2 trials, have investigated different target temperatures after out-of-hospital cardiac arrest [28, 29]. Based on the TTM and TTM 2 trials, the guidelines were subsequently updated to first recommend targeted temperature at 33–36 °C for 24 h followed by fever avoidance until 72 h [12] and latest to either a strategy of an initial target temperature for 33–36 °C followed by fever avoidance until 72 h, or solely fever avoidance for the total duration of 72 h [4]. The present investigation demonstrated no benefit on 1-year mortality from device-based fever prevention for 12 compared to 48 h after the initial period of temperature control at 36 °C for 24 h. Likewise, there was no benefit found for any of the predefined subgroups. This is consistent with the published results at 90 days [8]. Hypothermia is no longer regarded as a mandatory therapy after cardiac arrest by guidelines and as the BOX trial showed no difference according to 36 or 72 h of device-based fever prevention on both short and long-term outcomes, the question arises whether device-based fever avoidance should be reconsidered. The ongoing STEPCARE trial investigates this by randomizing out-of-hospital cardiac arrest patients to a strategy of fever prevention with and without device-based fever prevention.

Patients resuscitated from cardiac arrest but admitted to hospital still in a comatose state have a high risk of mortality during their initial hospital stay [1]. For survivors, the recovery process may be prolonged, some never recover completely, and their risk of mortality remains elevated after discharge [9–11, 30, 31]. The BOX trial found no treatment effects on short-term outcomes for a low compared to a high targeted blood pressure, a restrictive compared to liberal oxygenation target, or a shorter compared to longer duration of device-based fever prevention after cardiac arrest, and with the present investigation, we can also rule out any long-term effects on neurologic outcome or mortality [6–8]. These findings indicate that there were no major long-term differences in morbidity as a consequence of the interventions; however, we cannot rule out minor differences that might still be of patient relevance.

The BOX study solely included out-of-hospital cardiac arrest patients with a presumed cardiac cause of their arrest. Findings from the present investigation might therefore not be applicable for in-hospital cardiac arrest, or other than cardiac causes. For this investigation, neurologic outcome at 1 year was assessed based on medical records which should also be considered a limitation. Further, we cannot rule out that comparisons of even higher or lower blood pressure and oxygenation targets, or shorter or longer durations of device-based fever prevention would have yielded differentiated treatment effects.

## Conclusions

There were no indications of significant treatment effects on rates of mortality at 1 year of a low compared to high targeted blood pressure, a restrictive compared to liberal oxygenation target, or a shorter compared to longer duration of device-based fever prevention after out-of-hospital cardiac arrest. There was no sign of interaction between the three interventions. Nor were there indications of differentiated treatment effect according to predefined subgroups or for combinations of the randomizations.

### Supplementary Information

**Additional file 1. **Supplementary Appendix.

## Data Availability

Data availability will be subject to approval of the trial steering committee and relevant authorities following reasonable request.
