# Supplementary material for: Combined effects of targeted blood pressure, oxygenation, and duration of device-based fever prevention after out-of-hospital cardiac arrest on 1-year survival: post hoc analysis of a randomized controlled trial
Source: Crit Care. 2024 Jan 12;28:20. doi: 10.1186/s13054-023-04794-y (PMC10785348; doi:10.1186/s13054-023-04794-y)
Supplement: Supplementary file 1 — Additional file 1. Supplementary Appendix. [file 13054_2023_4794_MOESM1_ESM.pdf]

**Combined effects of targeted blood pressure, oxygenation, and duration of device-based fever prevention after out-of-hospital cardiac arrest on 1-year survival – Post hoc analysis of a randomized controlled trial**

SUPPLEMENT

Content:


In- and exclusion criteria for the BOX-Trial p 2

Forest plot for a low compared to high blood pressure target on mortality stratified by pre-defined subgroups p 3

Forest plot for a restrictive compared to liberal oxygen target on mortality stratified by pre-defined subgroups p 5

Forest plot for temperature control for 36 hours compared to 72 hours on mortality stratified by pre-defined subgroups p 7

Distribution of Cerebral Performance Category for a low compared to high blood pressure target at 1 year p 9

Distribution of Cerebral Performance Category for a restrictive compared to liberal oxygen target at 1 year p 10

Distribution of Cerebral Performance Category for temperature control for 36 hours compared to 72 hours at 1 year p 11


Citations in the supplement refers to the reference list in the main manuscript.

## In- and exclusion criteria for the BOX-Trial

| Inclusion criteria | Exclusion criteria |
|---|---|
| 1. Age ≥18 years<br>2. OHCA of presumed cardiac cause<br>3. Sustained ROSC (i.e. when signs of circulation persist and chest compressions have not been required for 20 consecutive minutes)<br>4. Unconsciousness (GCS < 8) (patients not able to obey verbal commands) after sustained ROSC | 1. Conscious patients (obeying verbal commands)<br>2. Females of childbearing potential (unless a negative HCG test can rule out pregnancy within the inclusion window)<br>3. In-hospital cardiac arrest<br>4. OHCA of presumed non-cardiac cause, e.g., after trauma or dissection/rupture of major artery OR cardiac arrest caused by initial hypoxia (i.e., drowning, suffocation, hanging)<br>5. Known bleeding diathesis (medically induced coagulopathy (e.g., warfarin, NOAC, clopidogrel) does not exclude the patient<br>6. Suspected or confirmed acute intracranial bleeding<br>7. Suspected or confirmed acute stroke<br>8. Unwitnessed asystole<br>9. Known limitations in therapy and Do Not Resuscitate-order<br>10. Known disease making 180 days survival unlikely<br>11. Known pre-arrest Cerebral Performance Category of 4<br>12. > 4 hours from return of spontaneous circulation to screening<br>13. Systolic blood pressure (SBP) < 80 mmHg despite fluid loading/vasopressor and/or inotropic medication/ intra-aortic balloon pump/axial flow device (If SBP is recovering during the inclusion window of 4 hours, the patient can be included)<br>14. Temperature on admission < 30 °C |

Modified from Kjaergaard et al., *N Engl J Med*. 2022; Schmidt et al., *N Engl J Med*. 2022; Hassager et al., *N Engl J Med*. 2022.[6-8]

# Supplemental Figure S1

| | High Blood Pressure Target | Low Blood Pressure Target | Hazard ratio (95% CI) |
|---|---|---|---|
| Overall: Low | 393 | 396 | 0.92 (0.72-1.16) |
| Male sex | 316 | 320 | 0.9 (0.69-1.17) |
| Female sex | 76 | 76 | 1 (0.61-1.64) |
| Above median age | 185 | 199 | 0.99 (0.74-1.33) |
| Below median age | 206 | 197 | 0.74 (0.5 -1.09) |
| Hypertension | 176 | 186 | 0.89 (0.64-1.23) |
| No hypertension | 215 | 210 | 0.96 (0.68-1.34) |
| Renal Impairment | 22 | 17 | 0.87 (0.37-2.05) |
| No renal impairment | 371 | 378 | 0.93 (0.72-1.18) |
| COPD | 30 | 33 | 1.78 (0.89-3.56) |
| No COPD | 362 | 362 | 0.84 (0.66-1.08) |
| Shockable rhythm | 356 | 350 | 0.88 (0.68-1.14) |
| Nonshockable rhythm | 35 | 46 | 0.9 (0.51-1.57) |
| STEMI | 172 | 178 | 0.98 (0.68-1.39) |
| No STEMI | 219 | 204 | 0.84 (0.61-1.15) |
| Site: RH | 250 | 253 | 0.83 (0.62-1.11) |
| Site: OUH | 143 | 143 | 1.09 (0.74-1.6 ) |

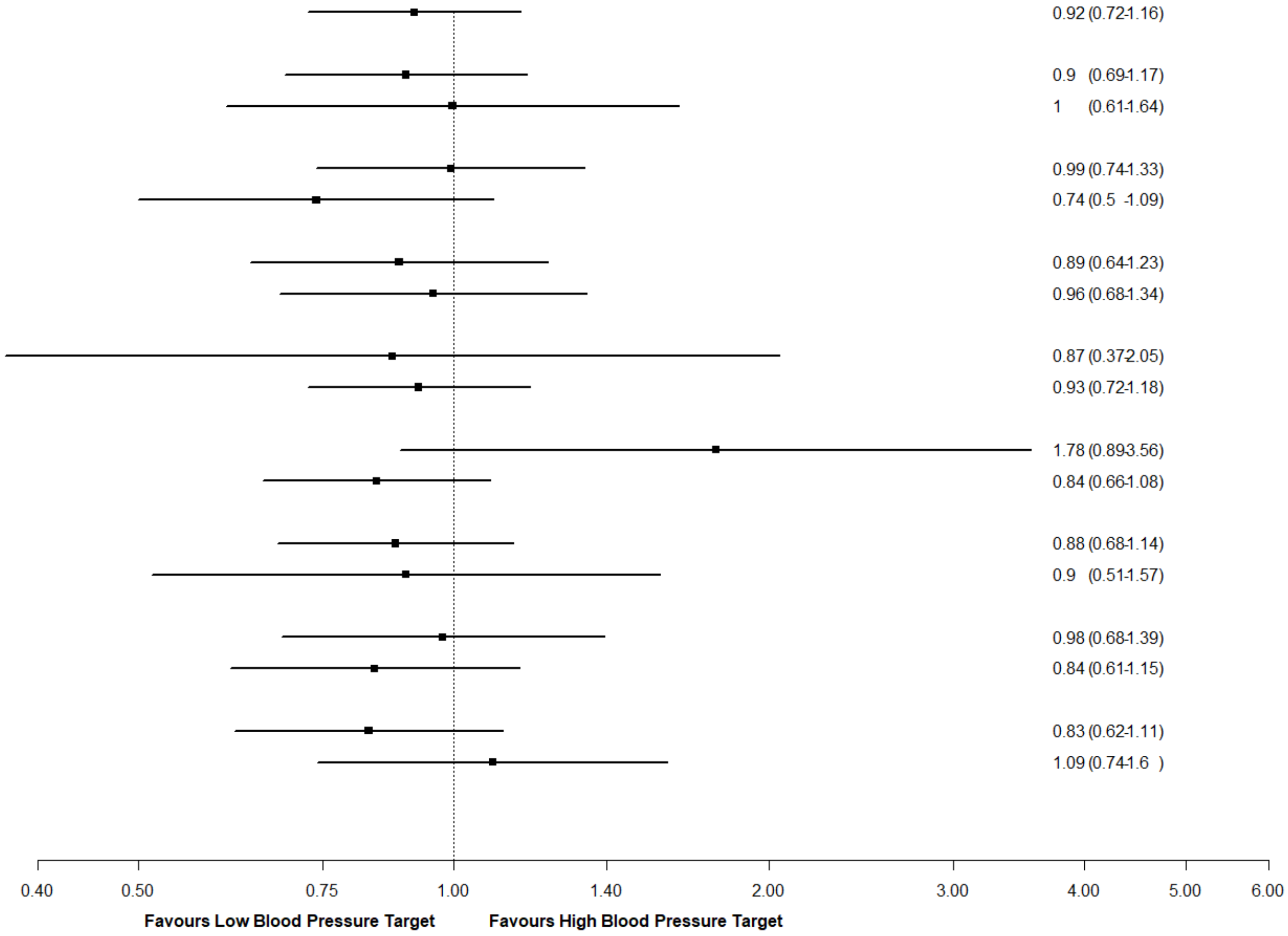

## Figure legend, Figure S1

Forest plot showing the effect of a low compared to high blood pressure target on mortality stratified by pre-defined subgroups. Hazard ratios indicate the hazard with a low blood pressure target. COPD denotes chronic obstructive pulmonary disease; STEMI, ST-segment elevation acute myocardial infarction; RH, Rigshospitalet, Copenhagen University Hospital, Denmark; OUH, Odense University Hospital, Denmark.

## Supplemental Figure S2

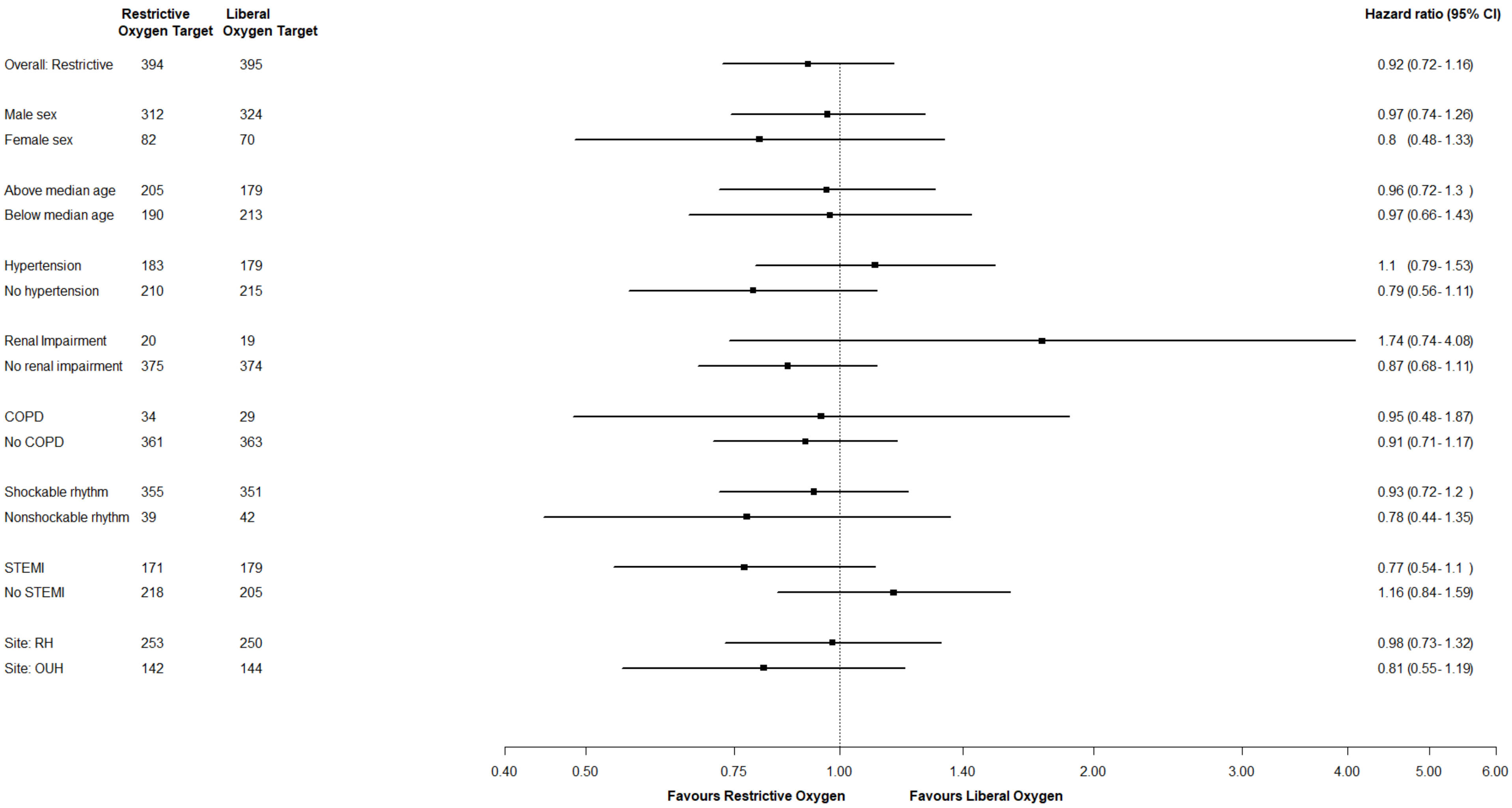

## Figure legend, Figure S2

Forest plot showing the effect of a restrictive compared to liberal oxygen target on mortality stratified by pre-defined subgroups. Hazard ratios indicate the hazard with a restrictive oxygen target. COPD denotes chronic obstructive pulmonary disease; STEMI, ST-segment elevation acute myocardial infarction; RH, Rigshospitalet, Copenhagen University Hospital, Denmark; OUH, Odense University Hospital, Denmark.

## Supplemental Figure S3

| | Temperature Control for 36 hours | Temperature Control for 72 hours | Hazard ratio (95% CI) |
|---|---|---|---|
| Overall: 36 hours | 393 | 396 | 0.98 (0.78-1.24) |
| Male sex | 320 | 316 | 1.08 (0.83-1.41) |
| Female sex | 73 | 79 | 0.72 (0.43-1.19) |
| Above median age | 205 | 197 | 0.95 (0.7 -1.27) |
| Below median age | 187 | 198 | 1.1 (0.75-1.62) |
| Hypertension | 186 | 176 | 0.83 (0.6 -1.15) |
| No hypertension | 202 | 216 | 1.25 (0.89-1.76) |
| Renal Impairment | 20 | 19 | 1.27 (0.55-2.95) |
| No renal impairment | 366 | 376 | 0.98 (0.77-1.25) |
| COPD | 26 | 37 | 0.81 (0.4 -1.61) |
| No COPD | 361 | 357 | 1.04 (0.81-1.34) |
| Shockable rhythm | 352 | 354 | 1.01 (0.78-1.31) |
| Nonshockable rhythm | 40 | 41 | 0.79 (0.45-1.37) |
| STEMI | 172 | 178 | 0.96 (0.67-1.36) |
| No STEMI | 216 | 207 | 1.04 (0.76-1.43) |
| Site: RH | 250 | 253 | 0.92 (0.68-1.23) |
| Site: OUH | 143 | 143 | 1.1 (0.75-1.61) |

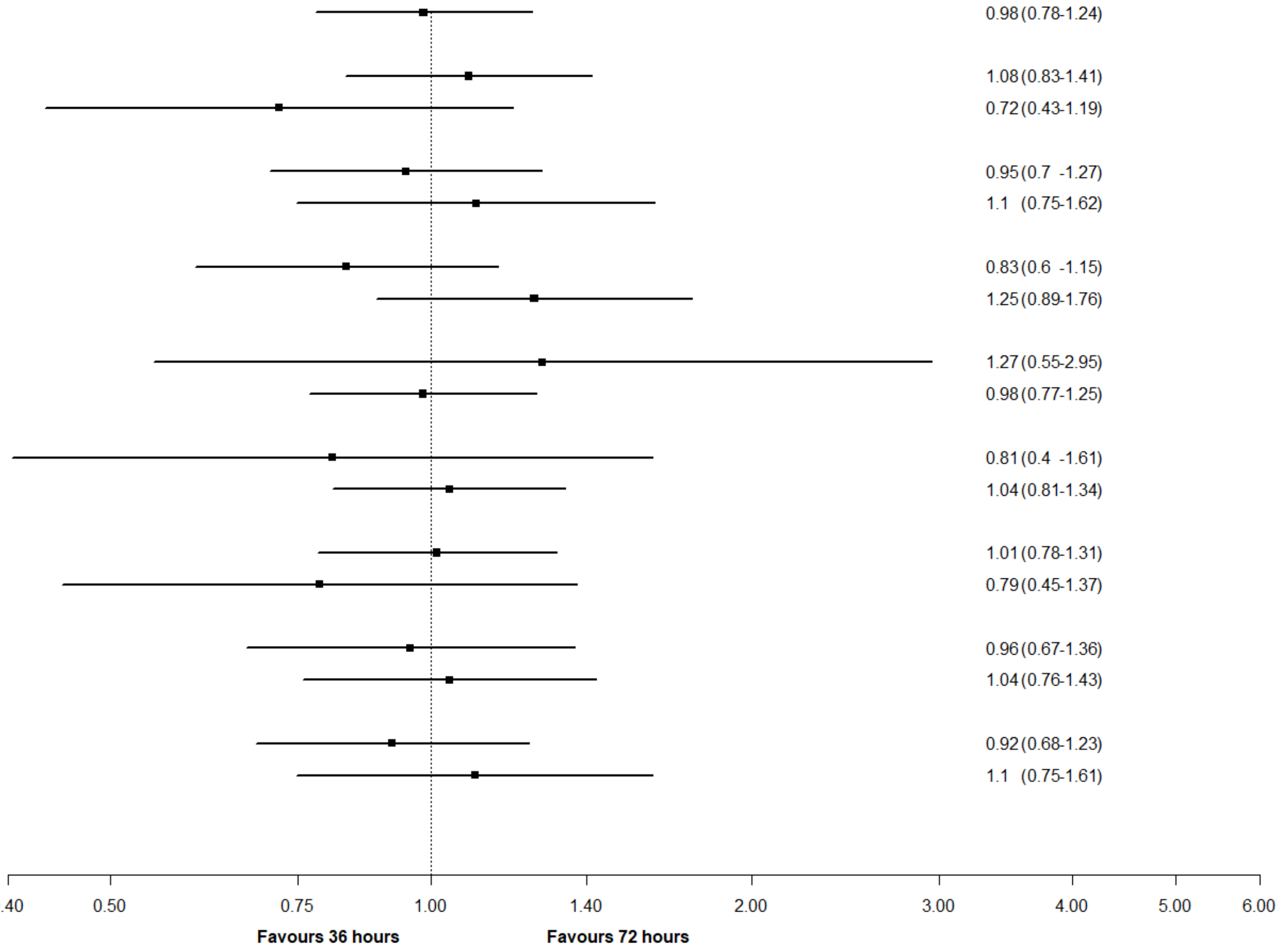

## Figure legend, Figure S3

Forest plot showing the effect of temperature control for 36 compared to 72 hours on mortality stratified by pre-defined subgroups. Hazard ratios indicate the hazard with temperature control for 36 hours. COPD denotes chronic obstructive pulmonary disease; STEMI, ST-segment elevation acute myocardial infarction; RH, Rigshospitalet, Copenhagen University Hospital, Denmark; OUH, Odense University Hospital, Denmark.

## Supplemental Figure S4

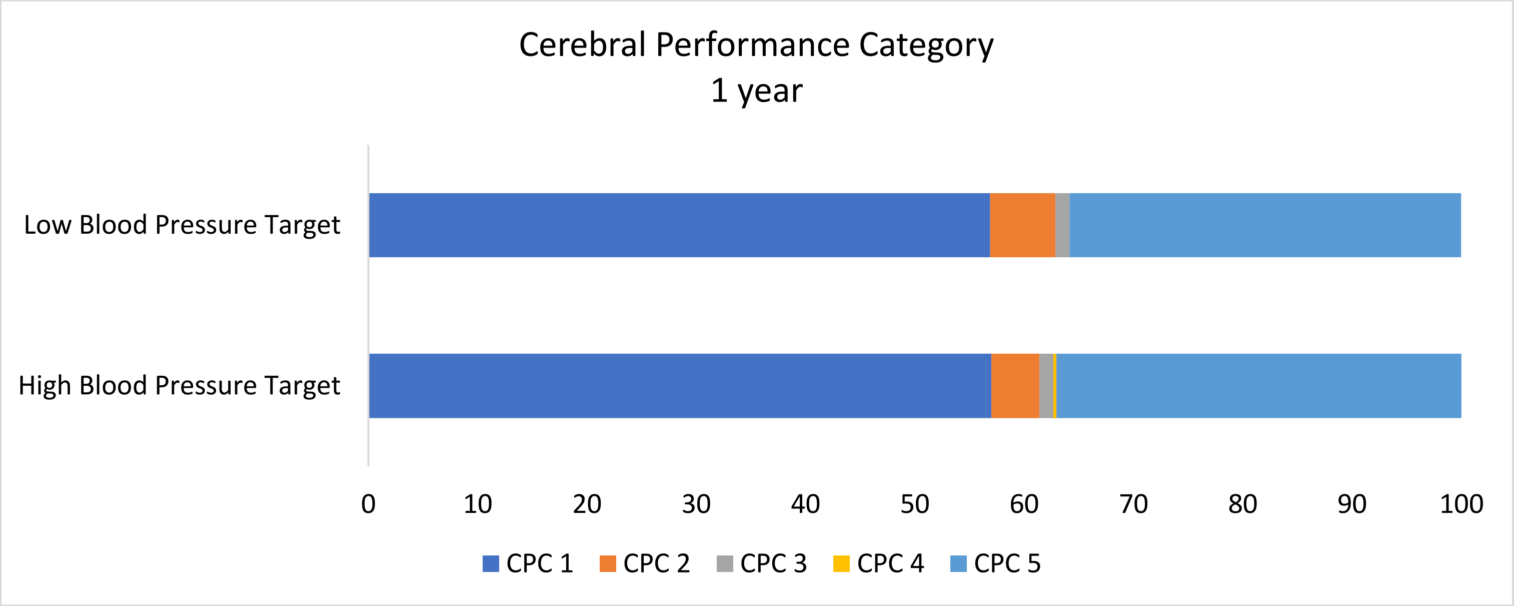


## Figure legend, Figure S4

Distribution of Cerebral Performance Category (CPC) for a low compared to high blood pressure target at 1 year. The CPC score ranges from 1 to 5 with lesser scores indicating better performance, and a score of 1 and 2 signifies a favorable neurologic outcome, with scores of 3 to 5 represents a poor neurologic outcome.[14-15]

Supplemental Figure S5

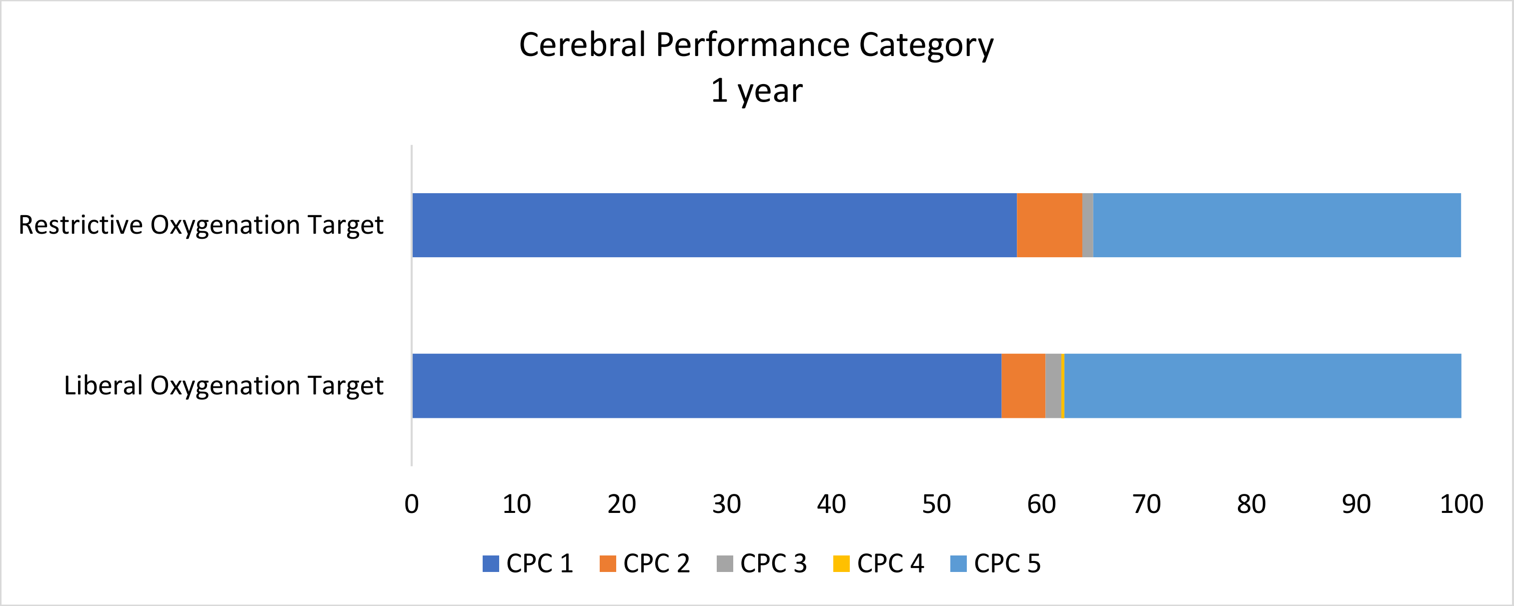


## Figure legend, Figure S5

Distribution of Cerebral Performance Category for a restrictive compared to liberal oxygen target at 1 year. The CPC score ranges from 1 to 5 with lesser scores indicating better performance, and a score of 1 and 2 signifies a favorable neurologic outcome, with scores of 3 to 5 represents a poor neurologic outcome. [14-15]

Supplemental Figure S6

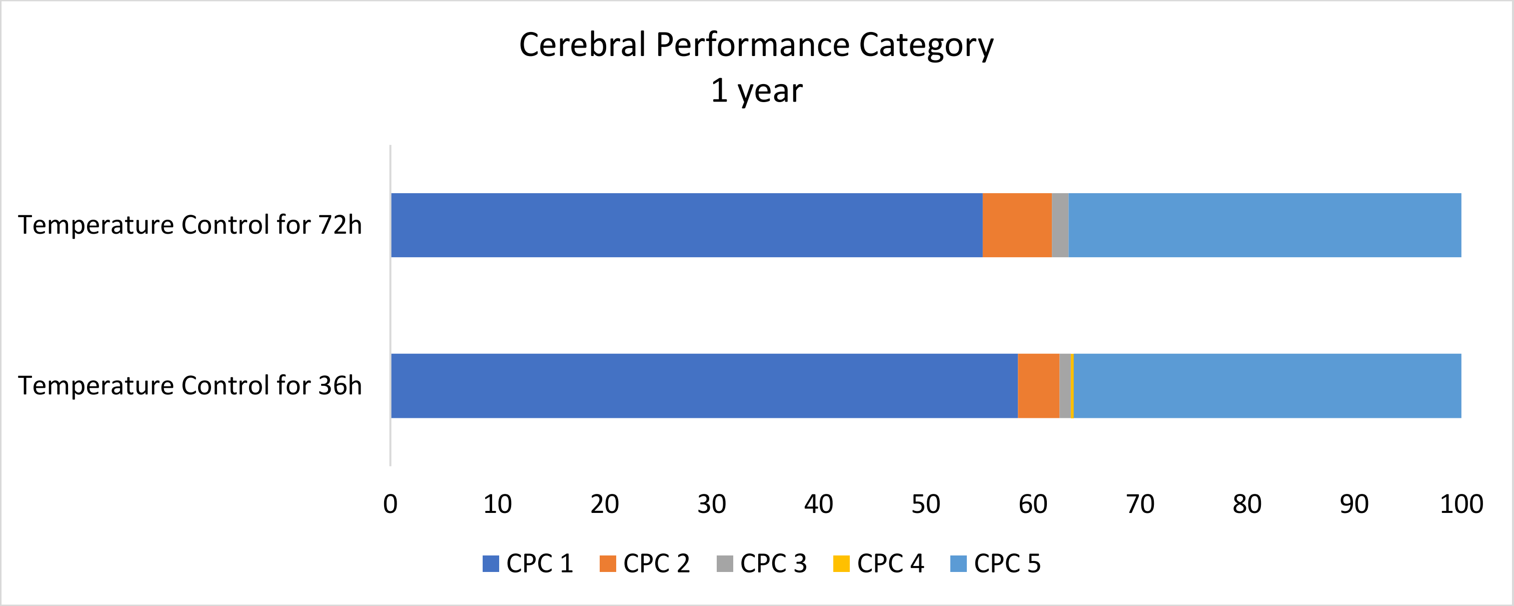


## Figure legend, Figure S6

Distribution of Cerebral Performance Category for temperature control for 36 hours compared to 72 hours at 1 year. The CPC score ranges from 1 to 5 with lesser scores indicating better performance, and a score of 1 and 2 signifies a favorable neurologic outcome, with scores of 3 to 5 represents a poor neurologic outcome. [14-15]
